# Punishing the individual or the group for norm violation

**DOI:** 10.12688/wellcomeopenres.15474.2

**Published:** 2020-02-13

**Authors:** Marwa El Zein, Chloe Seikus, Lee De-Wit, Bahador Bahrami

**Affiliations:** 1 Institute of Cognitive Neuroscience, University College London, London, WC1N 3AZ, UK; 2 Centre for Adaptive Rationality, Max Planck Centre for Human Development, Berlin, 14195, Germany; 3 Division of Psychology & Language Sciences, University College London, London, WC1H 0AP, UK; 4 Department of Psychology, Cambridge University, Cambridge, CB2 3EB, UK; 5 Faculty of Psychology and Educational Sciences, Ludwig Maximilian University, Munich, 80802, Germany; 6 Department of Psychology, Royal Holloway, University of London, Egham, Surrey, TW20 0EX, UK

**Keywords:** Social punishment, shared responsibility, group decisions, fairness, norm violations, individual differences

## Abstract

**Background:** It has recently been proposed that a key motivation for joining groups is the protection from consequences of negative behaviours, such as norm violations. Here we empirically test this claim by investigating whether cooperative decisions and the punishment of associated fairness-based norm violations are different in individuals vs. collectives in economic games.

**Methods:** In the ultimatum game, participants made or received offers that they could reject at a cost to their outcome, a form of social punishment. In the dictator game with third-party punishment, participants made offers to a receiver while being observed by a punisher, or could themselves punish unfair offers.

**Results: **Participants made lower offers when making their decision as part of a group as compared to alone. This difference correlated with participants’ overall mean offers: those who were generally less generous were even less so in a group, suggesting that the collective structure was compatible with their intention. Participants were slower when punishing vs not punishing an unfair offer. Importantly here, they were slower when deciding whether to punish or not to punish groups as compared to individuals, only when the offer concerned them directly in second party punishment. Participants thus take more time to punish others, and to make their mind on whether to punish or not when facing a group of proposers.

**Conclusions:** Together, these results show that people behave differently in a group, both in their willingness to share with others and in their punishment of norm violations. This could be explained by the fact that being in a collective structure allows to share responsibility with others, thereby protecting from negative consequences of norm violations.

## Introduction

To maintain individual and collective welfare, human society relies on formal and informal institutions of justice that enforce norms and punish norm violations. Punishing an individual for norm violations depends on whether they were the agent of that action and responsible for it ( Frith, 2014). To be protected against punishment, individuals delegate decisions to others, deferring responsibility and blame for an unfair behaviour ( Bartling & Fischbacher, 2012). An alternative way to shift the blame for an unfair choice is to share, rather than delegate, responsibility by making the decision in a group. Research on collective decisions has primarily focused on the benefits of group decisions in terms of outcome improvement, however, neglecting another facet: for an individual, being in a group could be a good way to reduce responsibility and thereby, the associated punishment for norm violation ( El Zein *et al.*, 2019). Performing an action as a group distributes the responsibility among group members and also makes it harder to determine who did what. When the group structure is not sufficiently transparent ( Duch *et al.*, 2011; Forsyth *et al.*, 2002; Gerstenberg & Lagnado, 2012), it seems likely that the severity of punishment for the collective as compared to the individual will decrease. Therefore, avoiding punishment may represent a strong motivation to join a group decision ( El Zein *et al.*, 2019).

*Indirect* empirical evidence supports this hypothesis that being in a group could help shift the blame and avoid punishments. People are more likely to display free-riding behaviours in groups ( Morgan & Tindale, 2002; Tindale & Kameda, 2017; Wildschut *et al.*, 2003), possibly thinking they might get away easier with their act as a group. Also, a group is judged less responsible ( Waytz & Young, 2012) and punished less severely ( Newheiser *et al.*, 2012) when perceived as a collection of distinct agents (low-cohesive group) than as a unified agent (high-cohesive group).

Here we aimed to *directly* test the hypothesis that norm violations and their punishments differ indecisions made alone or as a contribution to a group decision. Based on the hypothesis that shared responsibility in groups reduces punishment and blame ( El Zein *et al.*, 2019) we developed an experimental paradigm to test two key hypotheses: (1) Participants are more likely to violate norms when they are in a group. (2) For the same level of norm violation, groups are less likely (vs individuals) to receive punishment. To do so, we adapted well-known behavioural economic games, which provide valuable experimental paradigms to measure individual’s cooperative behaviours and responses to fairness-based norm violations. These games have repeatedly shown that humans cooperate with unrelated strangers in one-off encounters and bear personal costs to punish others who violate norms ( Fehr & Fischbacher, 2004). Previous studies have also identified important in-group biases in cooperative norm-enforcement in such games (for a review, see McAuliffe & Dunham, 2016). While the results are sometimes conflicting, an in-group preference is observed in both adults and children, suggesting that belonging to the same group may protect individual group members from punishment during cooperative interactions. Contrary to this line of research, our aim here is not to investigate how different group members interact with each other. We use a context where no group affiliation exists to explore how people behave if they are making cooperative decisions alone or as a part of a neutral group, and whether attitudes to norm-enforcement changes when cooperative decisions come from a neutral player vs a group of neutral players.

To do so, in our adapted versions of the ultimatum game (UG) and the dictator game with third-party punishment (TP-DG), individuals or groups of three individuals could split their allocated points with receivers. The group condition consisted of an average of offers and did not aim to account for an interactive collective decision. Rather, it accounted for individual behaviour in a context where participants were alone vs a context where individual choices contributed to a group average, making the final offer the responsibility of 3 rather than one person. In the UG, the receiver could reject an unfair offer which results in all players receiving zero points. This rejection is considered as a form of social punishment of the proposer and seems to reflect an emotional reaction ( Sanfey *et al.*, 2003) and signal of fairness needs ( Camerer & Thaler, 1995). In the TP-DG, a third-party can punish an unfair offer at their own cost. Even though unaffected by the norm violation, third parties display this cooperative behaviour which has been suggested to be driven by fairness needs similarly as in second-party punishment ( Fehr & Fischbacher, 2004).

 We note that while driven by our shared responsibility in groups hypothesis, our experimental design does not allow to characterize the exact mechanisms underlying differences in behaviour between norm violations and their punishment (and thereby be able to affirm that the effects are due to sharing responsibility solely relying on this experiment). However, to our knowledge, this is the first experimental design addressing whether these differences exist with such a controlled design, and directly comparing second and third-party punishment in repeated one-shot trials and a within-participant design.

 In addition to these two adapted games, we re-analysed available data from a previous study ( Rand *et al.*, 2009) that involved a public goods game between four players with punishment to test whether the use of punishment changes with the number of people defecting. Similarly to playing in a group, we hypothesize that when several people violate norms, they may get away easier with it under the shield that ‘others did it too’. This allows to share responsibility for a punishable act, which thereby may become less prone to punishment.

Applying our key two hypotheses described above to the experimental paradigm, we predicted that 1) an individual in the group will offer less than the same individual alone, and make his/her decision faster because of less hesitation about violating the norms within a group, 2) the group will be punished less than the individual and punishment vs no punishment decisions facing a group will be slower reflecting a more hesitant choice, and 3) inflicting punishment on unfair contributions will decrease with the number of people defecting. In an exploratory analysis, we collected self-reported scales to further investigate individual differences in norm violations and their punishment. Social value orientation was measured to link participants’ offering and punishment behaviours to a trait measurement of sharing with others ( Murphy *et al.*, 2011). A psychopathy scale ( Paulhus & Williams, 2002) was collected to test the idea that higher psychopathy scores may be associated with lower influence of being in a group or punishing a group, as people with higher psychopathy scores may care less about being in a group. Finally, political identification was measured in order to test whether differences would appear in fairness attitudes in the context of playing alone or as a group, because liberals and conservatives have been associated with different considerations of fairness and reciprocity ( Graham *et al.*, 2009).

## Methods

### Participants

A total of 150 healthy participants (79 females, mean age= 23.2±4.2) completed the experiment. The eligibility criteria were: 1) participants aged 18–35 and 2) have no reported history of neurological or psychiatric disorders. This sample size was decided based on a previous economic games study that we re-analysed here ( Rand *et al.*, 2009). The punishment treatment in the study included 40 participants. Given our 4 conditions of interest (individual or group proposers in the proposer or the punisher role), we multiplied this number by 4 and tested 150 (instead of 160) participants because of practical issues related to timing and participants recruitment. The study took place in November 2017 (first 80 participants – part of a master’s thesis of the second author; recruitment postponed for timing issues) and May 2018 (70 participants) at the Psychology Department testing cubicles (26 Bedford way, University College London (UCL). Participants were recruited through the UCL SONA Psychology Pool. It consists of a platform managed by UCL where the experimenter suggests experiment dates that participants receive by email and register to. Participants provided written consent according to regulations approved by the UCL ethics committee (Project ID Number: 4223/002 and ICN-AH-PWB-3-3-2016c). They were informed that they would receive £7.50 for their participation and could receive a bonus up to £2.5 based on their gains. All participants were accorded the bonus and compensated £10.

### Experimental design and procedure

**Figure 1.**
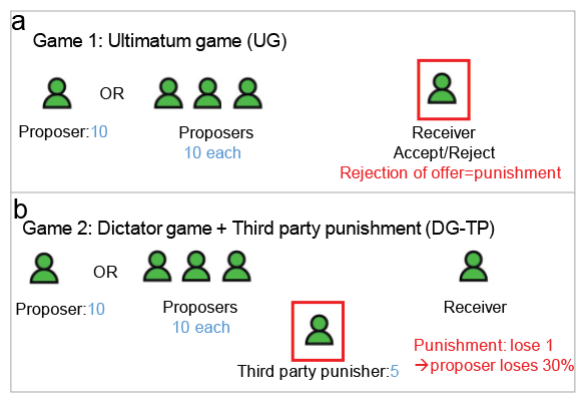
Experimental design. ( **a**, **b**) In both games, 1 proposer and a group of 3 proposers had to split points between themselves and a receiver. **a**) In the Ultimatum Game, the receiver could accept or reject the offer in which case no one received any points. **b**) In the dictator game with third party punishment, the receiver could not do anything, but a third-party punisher could punish the proposer(s) by making them lose 30% of their points at their own cost, i.e. losing 1 from their allocated 5 points.

Participants were recruited in groups of 7 to 11 individuals with mixed gender. They briefly met each other before entering separate cubicles to begin the experiment. After they completed practice trials, a message instructed them to wait for the experimenters to launch the experiments so that everyone started together. This setting was used to make participants believe they were playing together. The experiment was adapted from two well-known economic games: the Ultimatum Game (UG) and the Dictator game with third-party punishment (TP-DG) ( Figure 1).

***Ultimatum game (UG).*** This game includes 2 roles: the proposer and the receiver. In our rendition of the game, a proposer was given 10 points. S/he then decided how to split the 10 points between themselves and a receiver. The receiver, in turn, could accept or reject the offer. If accepted, each player received the points allocated to them by proposer. If rejected, both players received zero points. Rejection of an offer is a costly choice and is explained as a social punishment of the proposer by the receiver.

***Dictator game with third-party punishment (TP-DG).*** This game includes 3 roles: a proposer, a receiver and a third-party punisher. The proposer was initially given 10 points. S/he then decided how much of 10 points she wanted to give the receiver, and how much to keep. The third-party, who had been allocated 5 points, observed the transaction. S/he had the choice to spend one of her points to reduce the proposer’s overall outcome by 30%. The third party did not make any material gain from this choice. Reducing the proposer’s gain, therefore is a form of costly social punishment as the third-party loses a point in order to punish a player who acted unfairly.

***Key experimental conditions.*** In both games, we added a variation to the main paradigm to include conditions where groups (proposers) make the offers to the receiver. This condition consisted of a group of 3 individuals making a collective offer. Participants were informed that the group offer was an average of individual offers. They were told that punishment of the group offer would reduce each member’s pay-off directly and did not consist of a split of points among group members.

For example, in the UG, if the average group offer was 4, each member of the group kept 6 points if the offer was accepted. If the offer was rejected, everyone received zero points. In the TP-DG, if the average group offer was 4, each member of the group kept 6 points if the third-party did not punish them. If punished by third-party, each member of the proposer group received 4 points (i.e. 6 points reduced by 30% and rounded to nearest integer).

***Design.*** All participants completed 60 one-shot interaction trials in total and played all roles in both games. The trials were played anonymously assuring that participants could not build reputations that might influence their decisions. While participants were told they were playing online together, we computed all the interactions, and everyone did the same experiment (with randomized order of rounds for each participant).

Conditions of interest consisted of 48 trials, where participants played either receiver in UG (24 trials) deciding whether to accept or punish offers or the third-party punisher in TP-DG (24 trials). In these trials, offers were perceived to have been made by three individuals (group condition) on half of the times (12 trials) and by one individual (individual condition) in the other half. The participants did not know that these offers were algorithmically generated so that they ranged from 0 to 5 (each repeated twice within each individual and group condition) and therefore primarily consisting of unfair offers.

Participants also completed 12 trials in which they played the other roles. This included playing the proposer in the UG and TP-DG where they selected a number (out of ten, on the computer keyboard) to offer to the receiver. They played twice as an individual proposer and twice as a group of proposers in both games. They also played the receiver in the TP-DG in which they received an offer but could not respond (twice receiving the offer from an individual proposer and twice from a group of proposers). For these conditions, the other players’ choices were computed as follows: The proposers offers were randomly generated numbers between 0 and 6. The decisions to reject offers in UG or punish in TP-DG were based on the participant’s offer (or the mean offer with the other two simulated offers): if the offer was between 0 and 4, then there was a 50% chance it will get rejected/punished. If the offer was 5 or more then it was accepted/not punished.

Before starting the experiment, participants completed a practice with one round in each of the condition (five possible roles played in the group and individual condition – ten practice trials total).

***Trial structure.*** At each round, participants first saw which game they were paying for 5 seconds: the image depicted all the possible roles with the role they were assigned to on that round framed with a black rectangle. The points each player had was also reminded at each round. If they were in a group condition, three proposers appeared on the screen.

If they were playing the proposer role:

They were asked: *How much would you like to offer?* They could press a number on the keyboard to make their offer within 4 seconds. A spinner then appeared on the screen for ~5 seconds and it was written: *You offered* (or *you and the 2 other players offered* in the group condition) [amount offered], *the receiver* (UG) or *the punisher* (TP-DG) *is making a choice*. Then they saw what the receiver or punisher decided: ‘The receiver accepted’ or ‘rejected’/ ‘The proposer(s) was/were punished’

If they were playing the receiver role in UG or the third-party punisher:

They first saw a spinner for about ~5 sec and it was written: *The proposer is (or the 3 proposers are) making an offer.*

Second, the proposed offer was written: The proposer offered [amount offered].

Third, they were asked:

*Would you like to accept the offer?* (if receiver in UG) or *Would you like to punish the proposer?* (if punisher in TP-DG) They could press ‘Y’ for Yes or ‘N’ for No on the keyboard to give their answer. They had 4 seconds to make their choice.

If they were the receiver in the TP-DG, they observed what was happening, with spinners while proposer(s) made an offer and when the punisher was decide whether to punish or not, and the outcomes of each stage.

At the end of each round, participants were shown the outcomes for each player below the image depicting the player for 5 seconds (for example: The proposers each keep 6 – The receiver gets 4 – The punisher keeps 5)

The exact timeline of each round can be observed by following the link to the online experiment:

https://www.ucl.ac.uk/icn-crowd-cognition/Marwa/gamesexp/rungames.html

***Incentives.*** Participants were told that they would have the chance to win a bonus and receive up to an additional £2.5 on the basis of their outcome in a randomly selected trial at the end of the experiment (with 1 point=0.25pounds). This made sure that every trial counted for towards the participant’s earning and helped to make sure that they keep focused in all 60 trials.

***Questionnaires.*** Online questionnaires (using www.qualtrics.com/) were sent to the participants via email and filled out before the day of the experiment. Participants had to respond to these questionnaires in order to be eligible to participate in the experiment; however, they were not selected based on these scales in order to fit different groups. The questionnaires measured social value orientation (SVO) ( Murphy *et al.*, 2011), self-reported political identification (POI) (from extreme left to extreme right) and psychopathy traits extracted from The Dark Triad Scale ( Paulhus & Williams, 2002). We checked whether these three different scales co-varied with the four dependent variables: mean offers proposed OFF, mean punishment PUN, difference in offer between group and individual OFFDIFF, and difference in punishment given to a group vs individual PUNDIFF, and the associated reaction times (RTs). We also checked the relation between the scales and these variables separately in the UG and TP-DG.

***Statistical analyses.*** Analyses were performed using MATLAB (R2016b). Non-parametric analyses were performed as all data (offers made as proposers, proportion punishment and reaction times) were not normally distributed (Kolmogorov-Smirnov and Shapiro-Wilk tests rejecting the null hypothesis that the data come from a normal distribution). These analyses include the Wilcoxon signed-rank test, Friedman test, Spearman correlations and generalized linear mixed-effects models. Effect size (r) for Wilcoxon tests are reported, calculated as: r = Z/sqrt(N) with N = number of observations.

***Re-analysis of data from the public goods game.*** To investigate whether punishment use decreases with the number of defectors (3 ^rd^ prediction in the introduction) in the public goods game, we reanalyzed available data from a previous study ( Rand *et al.*, 2009) that involved a PGG between four players with punishment ( Figure 5a). We tested our third prediction that inflicting punishment on unfair contributions will decrease with the number of people defecting by examining the use of punishment at each played round as a function of the number of people defecting (rather than the number of people giving an offer as we did in our experimental setting). We considered as defectors the players who gave less than half of the maximum amount of contribution at each round (less than 10, maximum amount=20).

We performed a mixed model to test the hypothesis that punishment option (1 if any punishment is used, i.e., punishing 1 or more players, 0 if no punishment) was predicted by number of defectors. The number of defectors at the round, the player’s contribution and the group’s payoff were entered as fixed-effect predictors of punishment use, and participants were entered as random-effects (40 participants).

## Results

### Proposer role

Two independent variables could influence the offers made by proposers and reaction times to make the offers: the game (Ultimatum Game UG or Dictator Game with third party punishment DG-TP) and the group condition (Individual proposer IND or group proposer GRO).

***Proposer offers.*** Offers made in the UG correlated with those made in the DG-TP (ρ =0.60, p<0.001) confirming that people who are generous in one game were also generous in the other. Moreover, higher offers were made in UG as compared to DG-TP (Z = 3.86, p < 0.001, r = 0.22).

**Figure 2.**
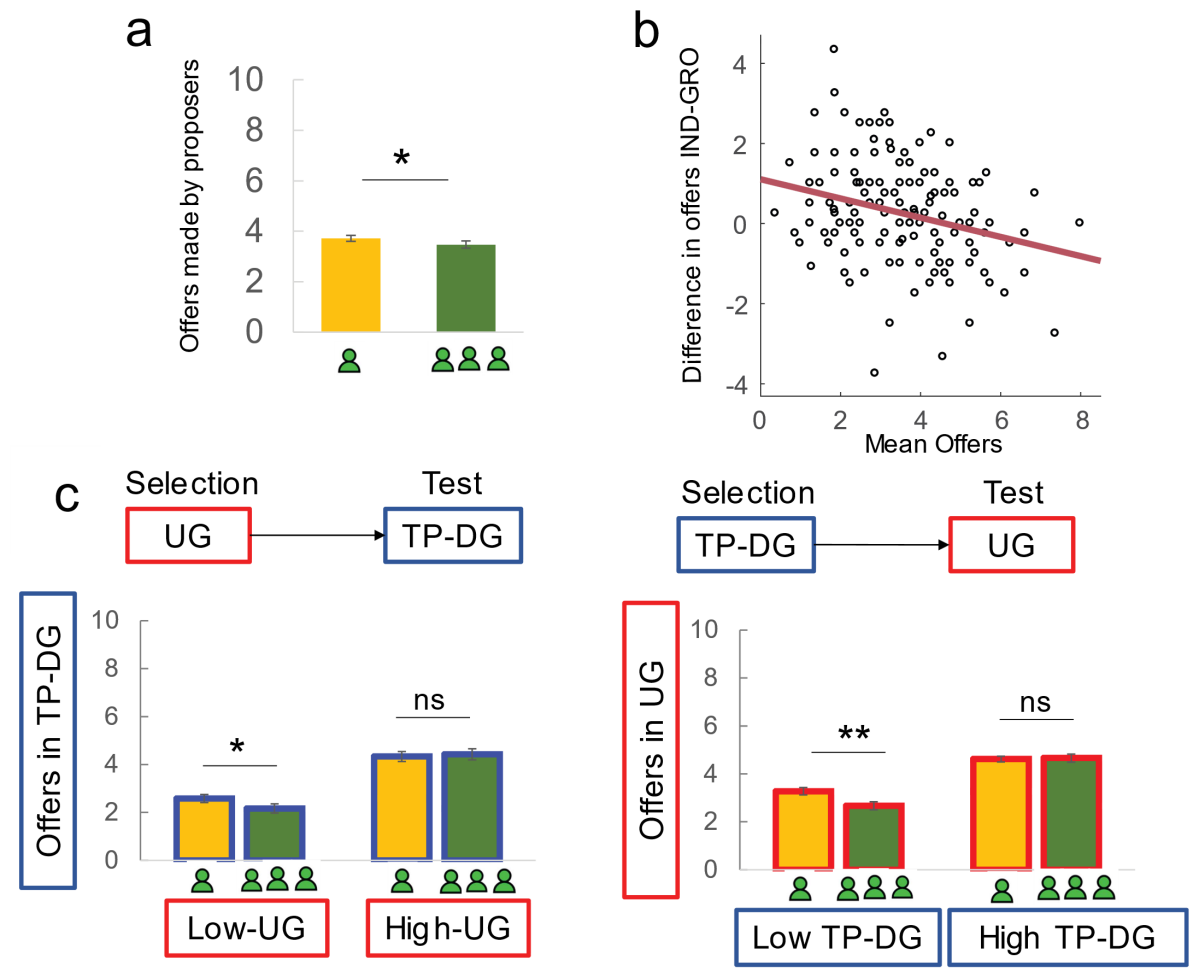
Offers made in the proposer role. ( **a**) Mean offers over both games made individually (yellow) or as a group (green). ( **b**) Difference in offers made individually or as a group as a function of mean offers over both games. ( **c**) Offers in each game as a function of a selection made in the other game: Left panel, offers in the third-party dictator game (TP-DG) separated by those who gave low or high offers in the Ultimatum Game (UG). Right panel, offers in the UG separated by those who gave low or high offers in the TP-DG. ** p<0.01, *p<0.05; ns, non-significant.

To test our first hypothesis, that an individual in the group will offer less than the same individual alone, we turn to the effect of group condition on offers. Confirming our hypothesis, a main effect of group condition was observed with higher offers made by participants as individual proposers (IND) as compared to being part of a group of proposers (GRO) (Z = 2.23, p = 0.025, r = 0.12) ( Figure 2a). This was also true when considering only the first trial where people made an offer individually and the first trial where they made the offer as a group average (Z=3.24, p=0.001, r=0.18). The difference between IND and GRO did not significantly differ between games (Z = 0.67, p = 0.49, r = 0.03). Interestingly, the offer difference between IND vs GRO correlated negatively with the mean offer made by each participant in all conditions (Spearman correlation ρ = -0.251, p = 0.002) ( Figure 2b). This correlation persisted within each game: In the UG, the difference between IND and GRO co-varied with the mean offer in UG (ρ = -0.31, p < 0.001) and the mean offer in TP-DG (ρ = -0.18, p = 0.02). In the TP-DG, the difference between IND and GRO co-varied with the mean offer in TP-DG (ρ = -0.19, p = 0.01) and the mean offer in UG (ρ = -0.16, p = 0.04). In other words, individuals who tended to make overall lower offers (regardless of the game played) diminished their offers even further when in a group, suggesting that the group condition was compatible with the individual’s intention to make less generous offers.

To further understand this finding, we categorized people as low proposers and high proposers using a median split in each of the games separately. Using the split based on the UG, we checked whether there was a difference between IND and GRO in low vs high proposers in the TP-DG: A significant effect appeared only for low proposers (Z = =2.04, p = 0.04, r =0.16), but not high proposers (Z =-0.61, p = 0.53, r =0.04, difference between the two types of proposers – low vs high proposer Z=1.9, p=0.05, r=0.15) ( Figure 2c). Similarly, using the split based on the TP-DG, we checked whether there was a difference between IND and GRO in low vs high proposers in the UG: A significant effect appeared only for low proposers (Z =3.16, p = 0.001, r =0.25), but not high proposers (Z =-0.23, p = 0.81, r =0.01, difference between the two types of proposers Z=2.83, p=0.004, r=0.23) ( Figure 2c).

***Reaction times to make offers.*** To test the second part of our first hypothesis that an individual in the group will make his/her decision faster as compared to the same individual alone, we turn to the differences in reaction times between the conditions. No main effect of game (p=0.18) or group (p=0.45) was observed. But there was an interaction between the two factors (Z = 2.72, p = 0.006, r=0.16): Reaction times were faster for decisions within a group as compared to individually only in the TP-DG (Z = -2.65, p = 0.008, r=0.15) and not in the UG (Z = -1.47, p = 0.14, r=0.08). The second part of our first hypothesis was confirmed, but only when third-party and not second party punishment was involved.

To conclude on the proposer role, participants gave lower offers as a group vs alone, and were faster to do so in the dictator game. This may suggest that participants were expecting less punishment when playing in a group as compared alone, which leads to our next question: Are groups punished less than individuals for the same norm violation and is the decision to punish or not punish a group as compared to one individual less intuitive?

### Punishment

Three independent variables could influence punishment: the amount of offers proposed (0 to 5), the game (UG or DG-TP) and the group condition (individual proposer IND or group proposer GRO).

**Figure 3.**
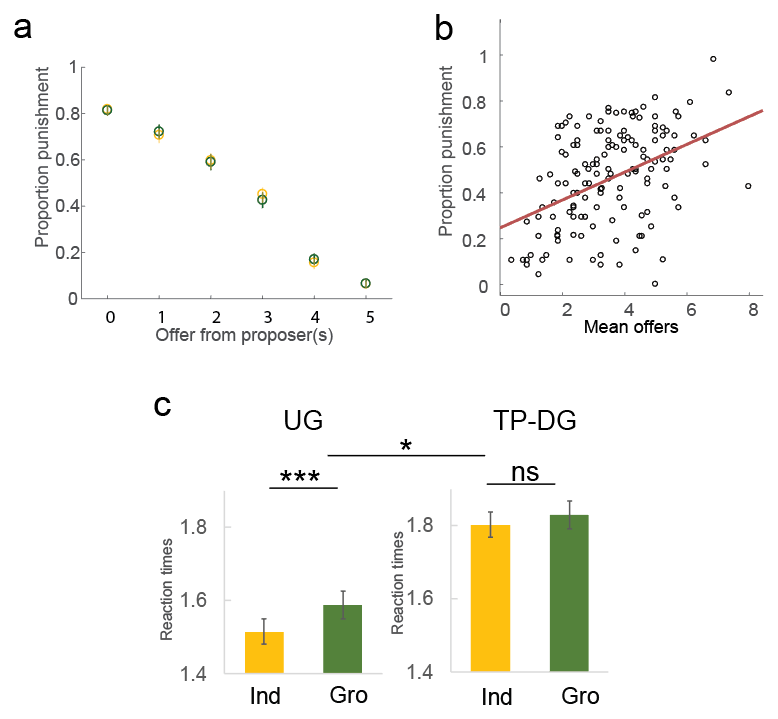
Punishment decisions. ( **a**) Proportion punishment as a function of the amount of offers proposed, green= for punishment of group, yellow= for punishment of individual. ( **b**) Proportion punishment as a function of mean offers. ( **c**) Reaction times for punishment vs no punishment decisions separated for the individual (yellow) and group (green) condition in the Ultimatum game (left panel, UG) and the third party punishment dictator game (right panel, TP-DG). *** p<0.001, * p<0.05; ns, non-significant.

***Proportion punishment.*** Proportion punishment in the UG correlated with proportion punishment in the DG-TP (ρ = 0.64, p < 0.001), suggesting that participants show a consistent pattern of punishment in different contexts, here second-party and third-party punishment. Proportion punishment also correlated with the amount of offers in the proposer role (ρ = 0.44, p < 0.001), showing that those who were more generous as proposers were also more prone to punish smaller offers, thereby caring about fairness both in their offers and in their punishment behavior ( Figure 3b). Similarly as for the proposed offers, there was more overall punishment in the UG than in the TP-DG (Z = 2.46, p = 0.014, r=0.14), consistent with a previous experiment directly comparing second and third-party punishment ( Fehr & Fischbacher, 2004).

A main effect of offers was observed, with punishment increasing as the offers decreased (Friedman test χ ^2^ =517.18, p < 0.001) ( Figure 3a). Contrary to our second hypothesis however, there was no main effect of group condition on proportion punishment (Z = 0.37, p = 0.7, r = 0.02) and no interaction between group and game.

***Reaction times for punishment decision.*** Confirming the second part of our second hypothesis, participants were slowed down to make their punishment vs no punishment decisions when facing a group of proposers as compared to an individual proposer (Z = 3.33, p=0.001, r=0.19). They were also slower to respond in the TP-DG as compared to the UG (Z = -9.33, p < 0.001, r=0.53). An interaction was observed between these group condition and games: the difference between reaction times for individuals vs groups was more important in the UG as compared to the TP-DG (Z = 1.96, p=0.04, r=0.11), with a significant difference between decision time for GRO vs IND only in UG (Z = 4.19, p < 0.001, r=0.24) and not in TP-DG (Z = 1.22, p = 0.22, r=0.07) ( Figure 3c). This shows that participants slowed down to make a decision when receiving an offer from a group vs an individual, only when they were directly receiving the offer. The slowing down to make a decision when facing a group vs individual proposer was true even for very low offers, i.e., 0 and 1 (Z = 1.98, p = 0.04, r=0.11), excluding the interpretation that slowing down is due to avoiding the punishment of fair participants trapped in a group with unfair partners as for offers of 0 and 1, every member of the group surely offered low amounts. Moreover, the difference in reaction times for IND vs GRO was only significant for punishment vs no punishment decisions of unfair offers (30% or less ( Sanfey *et al.*, 2003), so 0 to 3 here) and not fair offers (fair Z=1.1, p = 0.27, r=0.06; unfair Z= 3.43, p<0.001, r=0.2, difference Z=1.39, p=0.16, r=0.08). Participants were thus slowed down when it comes to punishing groups vs individuals who violated fairness norms.

***Reaction times as a function of punishment or no punishment decision.*** Participants were slower to punish as compared to not punish in both UG and TP-DG (Z = 4.02, p <0.001, r=0.23). This did not interact with the main effect of group on reaction times (Z=0.46, p=0.64, r=0.02). An interaction between the choice to punish or not to punish and the amount of offers proposed was observed: When the decision was to ‘not punish’, reaction times were slower for low (0, 1, 2) as compared to high (3, 4, 5) offers (Z = 4.74, p<0.001, r=0.27). When participants chose to ‘punish’, the reverse was observed as choices were faster for low offers vs high offers (Z = -3.07, p = 0.002, r=0.17).

### Individual differences

We accounted for the effects of all three scales on the different variables by entering them as predictors (SVO, psychopathy and political identification) of these variables in a generalized mixed model. This involved running 16 GLMs (8 variables OFF, PUN, OFFDIFF and PUNDIFF and associated RTs) X 2 games (UG and TP-DG). Although the 3 predictors were simultaneously entered in all GLMs, we present the results separately for each predictor for clarity. We provide the p values corrected for multiple comparison by multiplying the p values by the number of performed GLMs (16).

**Figure 4.**
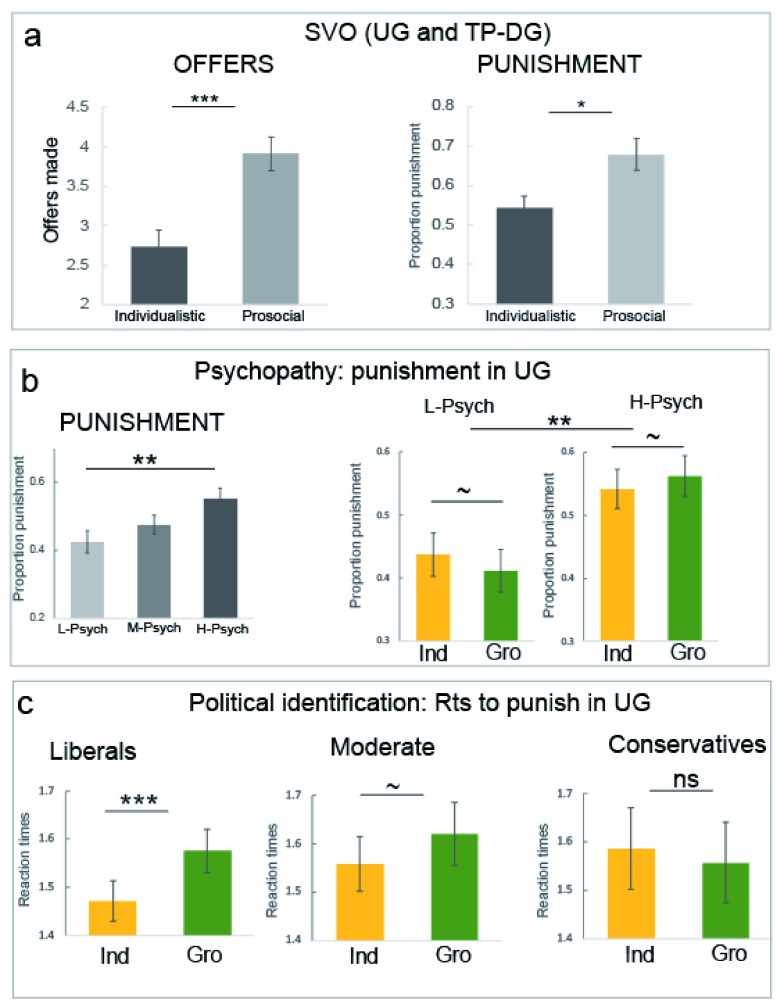
Individual differences. **a**) Social value orientation influence on offers (Left panel) and proportion punishment (Right panel). **b**) Psychopathy influence on proportion punishment in the ultimatum game. L-Psych: Low Psychopathy, M-Psych: Moderate psychopathy, H-Psych: High Psychopathy. An interaction was observed between Low and High psychopathy and the difference between individual and group punishment: For L-Psych, individuals are punished more than groups while for H-Psych, groups are punished more than individuals **c**) Political identification influence on reaction times for the punishment decision in the ultimatum game. Reaction times when faced with an individual or a group in liberals, moderate and conservatives., ~ p<0.06, *** p<0.001; ns, non-significant.

***SVO.*** SVO separates individuals in competitive, individualistic, prosocial, and altruistic profiles. On the total of 150 participants, 44 as individualistic, 105 scored as prosocial, and 1 as altruistic (Individualistic, prosocial and altruistic profiles were entered as 1, 2, 3 respectively in the GLMs). The amount of offers OFF was predicted by SVO (UG z = 4.38, p<0.001, corrected p<.01; TP-DG z=4.93, p<0.001, corrected p<.01). Punishment PUN was also predicted by SVO (UG z = 1.98 p=0.04, corrected p=0.64; TP-DG z = 2.76, p = 0.006, corrected p=0.096). Indeed, prosocials, compared to individualistics, gave higher offers as proposers (over both games Z=4.42, p<0.001, r=0.36; UG Z=3.66, p<0.001, r=0.29; TP-DG Z=4.32, p<0.001, r=0.35) and punished more as second and third-party punishers (over both games Z=2.08, p=0.03, r=0.16; UG Z=1.39, p=0.16, r=0.11; TP-DG Z=2.2, p=0.02, r=0.17) ( Figure 4a).

To conclude on SVO, people who have a generous trait gave higher offers in cooperative games. They also punished more unfair offers, however this last result was not robust enough to survive multiple comparisons.

***Psychopathy.*** PUN was predicted by psychopathy scores in the UG only (z = 2.37 p = 0.01, corrected p=0.16). Indeed, when participants were split into 3 (based on second and third quantile): high, moderate and low psychopathy, high psychopathy participants punished significantly more than Low psychopathy participants in the UG (Z=2.77, p=0.005, r=0.27 Figure 4b) and not in the TP-DG (Z = 0.41, p = 0.68, r=0.04).

Also, in the UG, the difference in punishment between groups and individuals PUNDIFF was predicted by psychopathy (z= -2.15, p = 0.03, corrected p=0.48): PUNDIFF significantly differed between Low and High psychopathy participants in the UG only (over both games Z=2.59, p = 0.009, r=0.25; UG Z = 2.78, p = 0.005, r=0.27; TP-DG Z=0.19, p=0.84, r=0.01) ( Figure 4b): In Low psychopathy, there was a higher proportion punishment of individuals as compared to groups (Z = 2.07, p = 0.03, r=0.20, UG Z=1.69, p=0.08, r=0.16; TP-DG Z=0.48, p=0.62, r=0.04). In High Psychopathy, there was no difference overall (Z=-1.27, p=0.2, r=0.12), but when only the UG was considered, it seemed like individuals were actually punished even less than groups (Z=-1.75, p=0.07, r=0.17).

The results thus show that high psychopathy participants rejected more offers overall and tend to do so more from groups than individuals. On the contrary, low psychopathy participants seem to reject more offers coming from individual as compared to group proposers. Again, these results are interesting but are to be taken with caution as the regressions results do not survive multiple comparison correction.

***Political identification.*** Political identification was measured on a scale from 1 to 7 from Strongly Liberal to Strongly Conservative (4=Neutral, entered this way in GLMs). In total, 80 participants identified as liberals, 19 as conservative and 51 as moderate. Difference in RT for punishing groups vs individuals was predicted by POI (z = 2.81, p = 0.005, corrected p=0.08).

Indeed, the observed slowing down for punishing groups was more important in liberals than in conservatives (UG Z=2.9, p = 0.003, r=0.29 TP-DG Z=-0.76, p=0.44, r=0.07). The difference in reaction times between punishing groups and individuals was the strongest in liberal participants (Z =-4.61, p<0.001, r=0.36; moderate participants Z=-1.84, p=0.06, r=0.18, conservatives Z=1.0, p=0.3, r=0.16) ( Figure 4c).

### Reanalysis of a public good game: Punishment as a function of the number of defectors

The results of our study show that only when participants are directly concerned by an offer, the number of people giving that offer influenced punishment behaviour: there is a consistent slowing down to make the decision of whether or not to punish three individuals as compared to one individual. We did not however find an effect on proportion punishment.

The UG involved punishment by rejecting an offer, the TP-DG involved a second step punishment that did not concern the third-party directly. A game that combines these 2 types punishment is the public goods game (PGG) with punishment, in which people can punish those who defect to a common good. In that case, people are directly concerned as they receive money from the common good (like in the UG) and they can decide to make a costly punishment at a second stage (like in the TP-DG).

**Figure 5.**
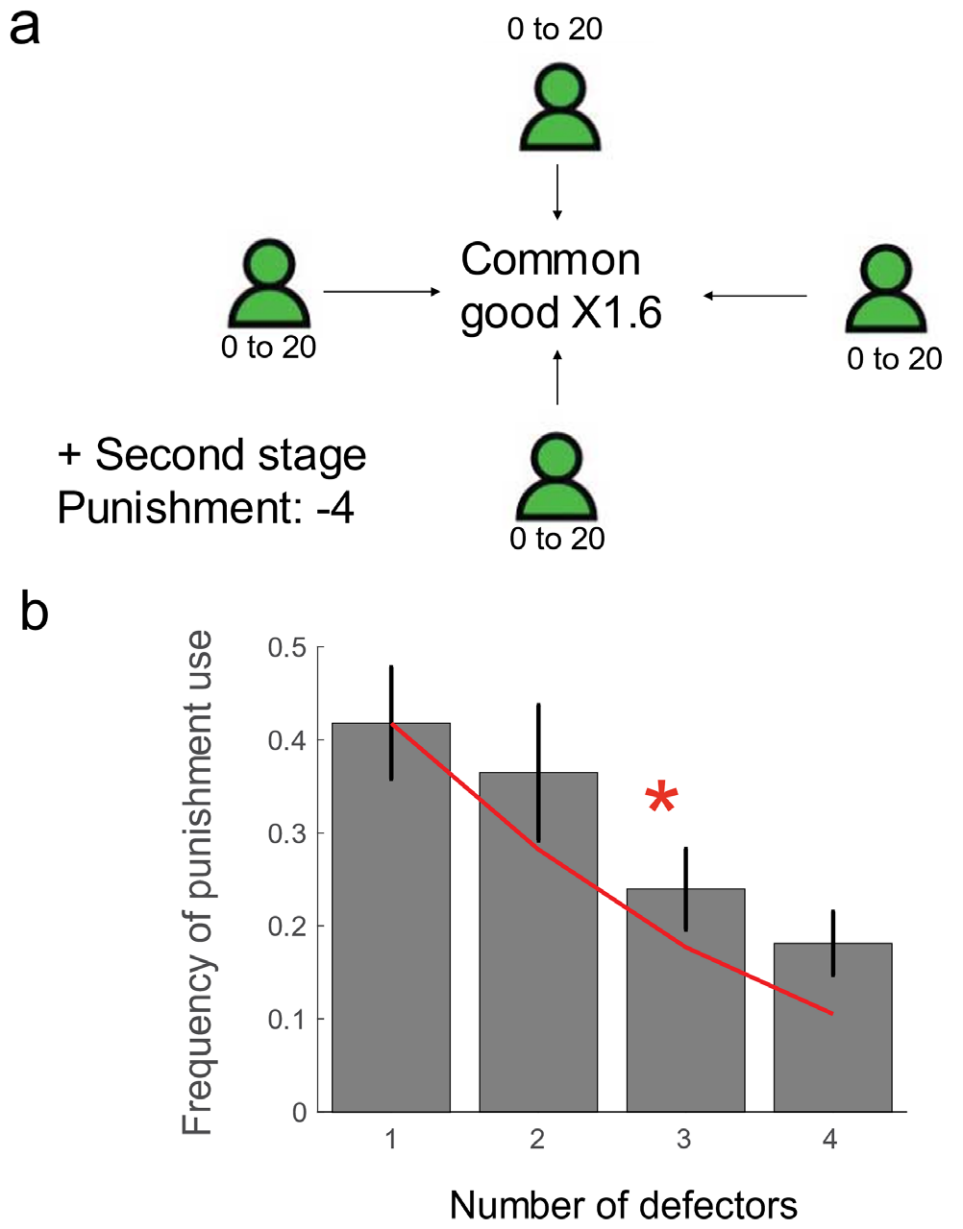
Re-analysis of a public good game with punishment. ( **a**) Structure of the public good games in Rand *et al.*, 2009: players can contribute to a common good from 0 to 20. The common good is multiplied by 1.6 and redistributed to all players. In a second stage, participants can punish others for their contributions by -12 at their own cost of losing 4. ( **b**) The frequency of using punishment as a function of the number of defectors. *p<0.05 significant decrease in the frequency of punishment use with the number of defectors in the mixed model.

To investigate what happens in such a context, we reanalyzed available data from a previous study ( Rand *et al.*, 2009) that involved a PGG between 4 players with punishment ( Figure 5a). We tested our third prediction that inflicting punishment on unfair contributions will decrease with the number of people defecting. The reasoning here is that the more defectors on a given round, i.e., the more people who violate the norm, the more their behaviour can be justified (they are not the only ones!) and can therefore benefit from reduced punishment. The number of defectors (from 1 to 4) decreased the probability of using punishment (Estimate=-0.504±0.22, Z=-2.20 p=0.02, no=516), even when accounting for the group’s payoff and the players’ contribution ( Figure 5b).

## Discussion

In this paper, we investigated whether norm violations and their punishments differ when made alone or as a group. We predicted that being in a group can shift the blame and punishment away from the individual because of shared responsibility for norm violations in a group. Our results confirmed our prediction in three ways: 1) Participants gave less generous offers (violated more the norm) when playing alone vs in a group of three. They were also faster to do so in the TP-DG. 2) Punishing a group vs an individual for norm violations required more time as participants were slowed down to make the punishment vs non-punishment decision. This was the case only in the UG with second-party punishment, when offers directly concerned the punisher. 3) Participants were less inclined to punish others for norm violations when the number of people committing these norms violations was high.

### Less generous offers in the group

Our current finding that people are less generous in a group corroborates the idea that people in groups violate the norms more than when alone given that (1) it replicates previous studies showing that individuals in groups display free-riding behaviours ( Morgan & Tindale, 2002; Tindale & Kameda, 2017; Wildschut *et al.*, 2003). These previous studies compared groups facing groups to individuals facing individuals and showed that groups are more competitive ( Wildschut *et al.*, 2003), defect more in a prisoner dilemma game ( Morgan & Tindale, 2002) and offer less in a joint decision in an ultimatum game ( Bornstein & Yaniv, 1998). Our results complement these studies by showing that even when facing one individual, people are less generous if they are part of a group vs alone. (2) Interestingly, here, we show for the first time that this decreased generosity in group correlates with people’s overall generosity. Indeed, only those who gave low offers displayed a difference between playing in a group or alone. This shows that the group was compatible with the intention of those who were less sensitive to the norms and violated them more. (3) It has been suggested that increased defection in groups may relate to reduced ‘identifiability’ as a group, supporting the idea that people feel less ‘accountable’ when making selfish choices ( Kugler *et al.*, 2012). Backing this, the fact that groups of two members that could be easily identified playing a dictator game gave higher offers as a group ( Cason & Mui, 1997) was linked to a reduced anonymity in such a context ( Luhan *et al.*, 2009). On the contrary, when anonymity is preserved in computer-based rather than face-to-face interactions, group members defect more than individuals. (4) Finally, groups that are procedurally interdependent ( Wildschut *et al.*, 2001) are less cooperative. This may be due to the fact that individual choices could not have been traced back, again allowing for anonymity and hiding behind the group ( Kugler *et al.*, 2012). Despite substantial evidence that the effects observed in our study may be due to increased norm violation in groups because of shared responsibility, our experimental design does not allow us to disentangle the exact mechanism(s) underlying the observed reduction of offers in groups, and other explanations may as well be possible. For example, people may become more rational ( Bornstein & Yaniv, 1998), and less biased as a group. They also may feel supported enough to act in a selfish, i.e. more greedy way ( Schopler *et al.*, 1995). In addition, one may argue that in our study, participants may have been basing their decision on the averaging procedure, by adjusting their offers to the expectations that others in the group will do. For example, a person who wants to give a low offer may lower their offer to offset the other group members offers. However, this last explanation is not consistent with the fact that only low offerors showed a difference between playing alone or in group as one would also expect those who want to give high offers to then adjust their behaviour by giving higher offers in groups (while they don’t). Finally, another possible explanation is that participants lowered their offers because of their expectation that other group members will also give low offers, given that predominantly unfair offers were presented in the context of the experiment (when participants were playing the receiver in the UG and the punisher in the TP-DG). However, the offer decrease for groups was already significant in the first round played in group as compared to the first round played individually, when they had less time to learn about the context they were playing in.

### Amount of punishment for groups vs individuals

Previous studies using economic games investigating punishment behaviours in groups have looked at how a group vs an individual punishes norm violations. They showed that when acting as third-party punishers in groups or alone, groups punish less severely in response to norm violations because of the diffusion of responsibility ( Feng *et al.*, 2016). In the present study, we examined how a group vs an individual is punished for norm violations rather than how the group punishes others. Contrary to our prediction that shared responsibility will also decrease the punishment of a group, we did not find any difference between the punishments of norm violations made by an individual vs a group. However, and in line with our prediction in our re-analysis of the public goods game, we did find evidence for decreased punishment with the numbers of defectors violating norms. Previous work has shown that a group is judged less responsible ( Waytz & Young, 2012) and punished less severely ( Newheiser *et al.*, 2012) when perceived as a collection of distinct agents (low-cohesive group) than as a unified agent (high-cohesive group). An explanation to the discrepancy in results could therefore be that in the public goods game, other players were perceived as a collection of individuals. On the contrary, in the current adapted version of the UG and TP-DG the group was possibly perceived as an entity as participants always saw the three group members when faced with the group and told that they can punish ‘the group’ rather than an individual in the group. Another possible reason why we did not observe decreased punishment for groups vs individuals in the context of our experimental design is linked to the effectiveness of the individual vs group punishment. Indeed, in the group condition, punishing the group imposed three times the total cost imposed when punishing in the individual condition. This implies that punishing a group was more effective than punishing an individual and one could then expect that the group should be punished more than the individual. This could have then overridden a decreased punishment of the group because of shared responsibility for low offers, possibly explaining why we did not observe any difference in the punishment of groups vs individuals.

### Decision time for punishing vs not punishing

We found that people were slowed down to punish as compared to not punish others for their norm violations. This suggests that punishing is less intuitive than not punishing. It relates to a series of discussions on whether the selfish (here not punishing) or the cooperative option (here punishing) is less of the default option for people. While some studies suggest that as observed here, it is less intuitive to choose the cooperative vs the selfish option ( Krajbich *et al.*, 2015), others suggest the opposite ( Rand *et al.*, 2012). These discussions were related to amounts of contributions in economic games (cooperative as high contributions and selfish as low contributions). Here we extend the discussion to punishment decisions, and show that in the context of a UG and TP-DG, people are slower to choose the punishment (and more cooperative) option. We importantly found that the punishment vs no punishment decisions were slower when punishers were faced with groups vs individuals, suggesting that it is also less intuitive to choose whether to punish or not to punish a group. This is in line with previous findings in an ultimatum game showing that participants spent less time considering whether to punish or not offers from opposite race as compared to same race ( Kubota *et al.*, 2013). Possibly, being faced with an individual vs group also made decisions faster because of a lower group affiliation when facing an individual vs a group. It is important to note that this effect was only present in second-party and not third-party punishment, suggesting that it applies only if unfairness is directed toward the self. Participants generally showed more punishment in second-party vs third-party punishment, reflecting a higher emotional response when being directly involved which may entail stronger inequity aversion and a higher need for fairness signalling ( Fehr & Fischbacher, 2004; Nowak *et al.*, 2000). This higher emotional involvement could also explain why the sensitivity to the group was higher in second-party vs third-party punishment.

### Social and antisocial punishment

The amount of punishment was predicted by both social value orientation and psychopathy scale in the ultimatum game (although should be taken with caution as the regression results did not survive multiple comparison corrections, but post-hoc analyses showed significantly higher punishment in both prosocials and high psychopathy participants). This could at first glance seem contradictory. Punishment consists of a cooperative option as it incurs a cost on the punisher, which explains why prosocial, compared to individualistic participants (as assessed in the social value orientation test), showed higher punishment rates in both second and third-party punishment. Interestingly, only in second-party punishment, proportion punishment also increased with the psychopathy scale. In the ultimatum game, punishment decisions have been associated with emotional reactions associated with anger ( Pillutla & Murnighan, 1996). Higher punishment in higher psychopathy participants could thus be associated to increased emotional reaction and an antisocial rather than prosocial reaction. This is line with the suggestion that in second-party, not third-party punishment, the decision to punish need not to reflect only cooperative behaviours but can also be associated with antisocial spiteful motives ( Jensen, 2010). Accordingly, our results also show that higher psychopathy is associated with higher punishment of the group vs the individual, while on the contrary the group benefited from lower punishment by low psychopathy participants, as initially predicted by our shared responsibility hypothesis ( El Zein *et al.*, 2019).

To conclude, using cooperation economic games, we show that people’s attitudes related to norm violations are influenced by whether they were made by an individual or a group. People are less generous as a group, use less punishment when more people defect the norms, and take more time to punish a group vs an individual who behaves unfairly to them. Together, these results support the idea that being part of a group may protect one from punishments and norm violations, possibly because of shared responsibility among group members for the same acts that can reduce blame and punishments ( El Zein & Bahrami, 2019; El Zein *et al.*, 2019).

## Data availability

Open Science Framework: Supplemental materials for preprint: Punishing the individual or the group for norm violation. https://doi.org/10.17605/OSF.IO/HPVBG ( El Zein, 2019).

This project contains the following underlying data:

- Data_punishment.csv (data for each task performed by each participant; a data dictionary is available in the Description).

Data are available under the terms of the Creative Commons Zero "No rights reserved" data waiver (CC0 1.0 Public domain dedication).
