# Supplementary material for: Punishing the individual or the group for norm violation
Source: Wellcome Open Res. 2020 Feb 13;4:139. Originally published 2019 Sep 20. [Version 2] doi: 10.12688/wellcomeopenres.15474.2 (PMC7062205; doi:10.12688/wellcomeopenres.15474.2)
Supplement: Peer review history

# Peer review: Reviewer response for version 2

**DOI:** 10.21956/wellcomeopenres.17253.r37911

**Published:** 2020-03-06

**Authors:** Justin W. Martin

**Affiliations:** 1 Department of Psychology, Boston College, Chestnut Hill, MA, USA

I thank the authors for their detailed responses to the concerns/issues I raised about the first version of this manuscript. At this point, I have no further reservations about this work.

I confirm that I have read this submission and believe that I have an appropriate level of expertise to confirm that it is of an acceptable scientific standard.

# Peer review: Reviewer response for version 2

**DOI:** 10.21956/wellcomeopenres.17253.r37910

**Published:** 2020-02-20

**Authors:** Yarrow Dunham

**Affiliations:** 1 Department of Psychology, Yale University, New Haven, CT, USA

The authors have satisfactorily responded to my comments.

I confirm that I have read this submission and believe that I have an appropriate level of expertise to confirm that it is of an acceptable scientific standard.

# Peer review: Reviewer response for version 1

**DOI:** 10.21956/wellcomeopenres.16925.r37132

**Published:** 2019-11-26

**Authors:** Yarrow Dunham

**Affiliations:** 1 Department of Psychology, Yale University, New Haven, CT, USA

An interesting take on the role of individual decisions made in the context of individuals or the contexts of a collective. I had several comments that I hope will help the authors as they go forward in this work.

 I don’t think the decision that individuals make in the collective condition is really a “collective decision” in that the individual decisions are just summed; something like a voting procedure to determine the decision would feel collective; this feels more like an individual decision in a collective context. I think making this distinction clearer would be useful.

 The introduction might want to do an earlier and clearer job distinguishing the present work from the literature on punishment of individuals who are identified as members of groups, i.e. on ingroup bias in punishment decisions.

 One interesting point that distinguishes the two conditions in these studies is that punishment or rejection is more effective in the group contexts because it imposes 3x the total cost; what impact, if any, do the authors think this could have?

 Th offers that were responded to ranged for 0 to 5 of 10, each repeated twice, in each game. As the authors note this means they were mostly unfair offers. Could this have affected responses? That is, over the course of the task responders and punishers are learning that the offers tend to be low, meaning they are learning something about the context of cooperation (or lack thereof). Could the authors do any analyses of trial effects to see if punishment and/or rejection increases in frequency over time?

 I also wonder about the potential role of expectation. If I am in a collective context and I think that others in my collective are likely to give low offers, what effect, if any, might this have on my offers? Is it possible I would lower my own offer as a result of the expectation that my group will make generally low offers? If so, this isn’t diffusion of responsibility or free-riding in the classic sense.

 Is Fig 3b correct? Why does punishment positively correlate with mean offer? Shouldn’t it be the other way around?

 The authors should justify why they chose the particular individual difference measures that they included. This was not well stated or established in the paper. Why these and not any of the many other measures related to group psychology? Also these measures involved analyses; were any of these predictions outlined in advance and so confirmatory tests? If not perhaps some sort of p-value correction would be needed to avoid spurious findings?

 In terms of the Rand re-analysis, what do the authors make of the fact that the difference did not appear between 1 and 2 defectors? More generally the logic of including the Rand re-analysis in this paper wasn’t as clear as it could be; more discussion of what it adds would be helpful.

I confirm that I have read this submission and believe that I have an appropriate level of expertise to confirm that it is of an acceptable scientific standard, however I have significant reservations, as outlined above.

# Response

**Published:** 2020-02-07

**Authors:** Marwa El Zein

**Affiliations:** University College London, UK

*An interesting take on the role of individual decisions made in the context of individuals or the contexts of a collective.*

**

We thank the reviewer for noting the interest of our work.

*I had several comments that I hope will help the authors as they go forward in this work. I don’t think the decision that individuals make in the collective condition is really a “collective decision” in that the individual decisions are just summed; something like a voting procedure to determine the decision would feel collective; this feels more like an individual decision in a collective context. I think making this distinction clearer would be useful.*

We agree with the reviewer and have changed our vocabulary throughout the text, to clarify that we are investigating an individual decision in a collective context rather than assessing the emergence of a collective decision. We avoided any use of ‘collective decision’, which we replaced by ‘making decision as part of a group’. We clarify since the introduction that it is a group average that we use in our design:

 ‘Here we aimed to *directly* test the hypothesis that norm violations and their punishments differ in decisions made alone or as a contribution to a group decision.’

‘In our adapted versions of the ultimatum game (UG) and the dictator game with third-party punishment (TP-DG), individuals or groups of three individuals could split their allocated points with receivers. The group condition consisted of an average of offers and did not aim to account for an interactive collective decision. Rather, it accounted for individual behaviour in a context where participants were alone vs a context where individual choices contributed to a group average, making the final offer the responsibility of 3 rather than one person.’

*The introduction might want to do an earlier and clearer job distinguishing the present work from the literature on punishment of individuals who are identified as members of groups, i.e. on ingroup bias in punishment decisions.*

We added this paragraph in the introduction to make the requested distinction:

‘These games have repeatedly shown that humans cooperate with unrelated strangers in one-off encounters and bear personal costs to punish others who violate norms ( Fehr & Fischbacher, 2004). Previous studies have also identified important in-group biases in cooperative norm-enforcement in such games (for a review, see McAuliffe & Dunham, 2016). While the results are sometimes conflicting, an in-group preference is observed in both adults and children, suggesting that belonging to the same group may protect individual group members from punishment during cooperative interactions. Contrary to this line of research, our aim here is not to investigate how different group members interact with each other. We use a context where no group affiliation exists to explore how people behave if they are making cooperative decisions alone or as a part of a neutral group, and whether attitudes to norm-enforcement changes when cooperative decisions come from a neutral player vs a group of neutral players.’

*One interesting point that distinguishes the two conditions in these studies is that punishment or rejection is more effective in the group contexts because it imposes 3x the total cost; what impact, if any, do the authors think this could have?*

**

We thank the reviewers for noting this important point that should be discussed. The point of using such a payoff was to keep the outcome similar from the perspective of the individual. This was at the expense that indeed punishing a group vs an individual imposes an overall 3X total cost, making the punishment more effective in the group condition. From this perspective, one could expect increased punishment in the group condition (because it is more effective). However, we did not observe any differences in the proportion punishment. One possibility is that:

1. There is higher punishment in the group condition because it more effective.
2. But the fact that it is group sharing responsibility for low offers, allows the group to benefit from reduced punishment (our hypothesis).

- These two points can cancel each other out so that no difference is observed between the individual and group condition. Future experiments would allow to test this explanation, for example, by using a similar design as ours, but precising that the group decision is an average of 2 previous participant’s offers (that will not incur any cost in the present experiment) and a current participant that would incur the cost of punishment. This would allow to judge a decision coming from a group of three, but impose a similar cost as in the individual condition. If in this scenario, the proportion punishment would decrease, then it would support the explanation offered here.

We added this discussion point to the manuscript:

‘Another possible reason why we did not observe decreased punishment for groups vs individuals in the context of our experimental design is linked to the effectiveness of the individual vs group punishment. Indeed, in the group condition, punishing the group imposed three times the total cost imposed when punishing in the individual condition. This implies that punishing a group was more effective than punishing an individual and one could then expect that the group should be punished more than the individual. This could have then overridden a decreased punishment of the group because of shared responsibility for low offers, possibly explaining why we did not observe any difference in the punishment of groups vs individuals.’

*The offers that were responded to ranged for 0 to 5 of 10, each repeated twice, in each game. As the authors note this means they were mostly unfair offers. Could this have affected responses? That is, over the course of the task responders and punishers are learning that the offers tend to be low, meaning they are learning something about the context of cooperation (or lack thereof). Could the authors do any analyses of trial effects to see if punishment and/or rejection increases in frequency over time?*

To answer the reviewer, we observed punishment behaviours over the course of time by splitting the rounds into 3:

Graph 1 represents the proportion punishment (or rejection, pooled or both games), pooled over individual and group conditions. A significant decrease in proportion punishment was observed in the third part of the experiment as compared to the second part ( Z=2.69, p=0.007). No other changes were significant.

When individual and group conditions were separated, it appeared that no changes in punishment behaviour were observed through time in the individual condition( Graph 2). On the contrary, there was a significant increase in group punishment in the second vs first part of the experiment (Z=-2.01, p=0.0439) and then a reversal with a significant decrease in punishment from part 2 to part 3 (Z=3.61, p<.001)( Graph 3).

When checking whether differences between individual and group punishment appeared in each of the parts separately, still no significant change was observed. Nevertheless it is worth noting that in part 2, there was a tendency for higher punishment of the group vs individuals ( Z=-1.67, p=0.0935) while the contrary tendency was observed in part 3 with higher punishment of individual vs group (Z=1.60, p=0.108). This somehow fits with the 2 opposite predictions from the effectiveness vs shared responsibility in group discussed above, and suggests that maybe people may use different strategies in how they punish groups vs individuals in different timepoints of the experiment. However, this would need further investigation and is just a discussion given the current presented results.

To go back to the reviewer’s point that participants may have learned that this is a context with low cooperation and increase their punishment over time, we seem to rather observe a decrease in punishment in the last part of the experiment. And this decrease is specific to the group punishment. It is possible to imagine that people increase the group punishment after observing in the first part of the experiment that offers are low (and the increase observed only in group because it is more effective to punish the group), but then give up on their norm-enforcement behaviour in the last part of the experiment. If this last decrease was observed in both group and individual conditions, it could have been solely explained by the motivation to maximize gains at this point of the experiment. However, the fact that the decrease is only in the group condition may suggest that they end up punishing less the group specifically because the group shares responsibility for the low offers that everyone seems to be giving in this context (decrease their norm-enforcement of a group specifically in a context where cooperation is low).

*I also wonder about the potential role of expectation. If I am in a collective context and I think that others in my collective are likely to give low offers, what effect, if any, might this have on my offers? Is it possible I would lower my own offer as a result of the expectation that my group will make generally low offers? If so, this isn’t diffusion of responsibility or free-riding in the classic sense.*

**

This is a very interesting and plausible explanation to the observed decrease in the group offers in the group condition. We attempt to test for whether it explains our results: We reasoned that the collective context of low cooperation should be first learned, before participants would lower their offers because everyone else is also giving low offers (so I expect others to give low offers and prefer to have a similar behaviour as everyone else). Therefore, if the participants give lower offers as group vs individual players since the first decision as offerors, then there is much less chance that they would have already learned the context of (lack of) cooperation. We compared the first offer as an individual player to the first offer as a group and found a significant decrease in the offer in the group vs individual condition (Z=3.24, p=0.0012), We added this analysis in the results:

‘This was also true when considering only the first trial where people made an offer individually and the first trial where they made the offer as a group average (Z=3.24, p=0.001, r=0.18).’

And in the discussion: ‘Finally, another explanation is that participants lowered their offers because of their expectation that other group members will also give low offers, given that mostly unfair offers were presented in the context of the experiment (when participants were playing the receiver in the UG and the punisher in the TP-DG). However, the offer decrease for groups was already significant in the first round played in group as compared to the first round played individually, when they had less time to learn about the context they were playing in.’

*Is Fig 3b correct? Why does punishment positively correlate with mean offer? Shouldn’t it be the other way around?*

Yes Fig 3b is correct indeed: punishment positively correlated with mean offer showing that people who are more generous, also care more about norm-enforcement, possibly to preserve a cooperative context. We agree with the reviewer that the opposite could have also been true so that people who are more generous don’t want to punish other. We believe this to relate to our discussion on social and antisocial punishment : one can expect punishment to be social because it has the good intention of establishing cooperative norms, but also punishment can be antisocial in the sense that is driven by an angry reaction toward an unfair offer and involves incurring a cost on others. Interesting we found that both prosocials and those who score high on psychopathy seem to show an increased punishment behaviour corroborating this idea of the co-existence of social and antisocial punishment.

*The authors should justify why they chose the particular individual difference measures that they included. This was not well stated or established in the paper. Why these and not any of the many other measures related to group psychology? Also these measures involved analyses; were any of these predictions outlined in advance and so confirmatory tests? If not perhaps some sort of p-value correction would be needed to avoid spurious findings?*

We now explain in the introduction why we decided to include these specific questionnaires:

‘In an exploratory analysis, we collected self-reported scales to further investigate individual differences in norm violations and their punishment. Social value orientation was measured to link participants’ offering and punishment behaviours to a trait measurement of sharing with others ( Murphy et al., 2011). A psychopathy scale ( Paulhus & Williams, 2002) was collected to test the idea that higher psychopathy scores may be associated with lower influence of being in a group or punishing a group, as people with higher psychopathy scores may care less about being in a group. Finally, political identification was measured in order to test whether differences would appear in fairness attitudes in the context of playing alone or as a group, because liberals and conservatives have been associated with different considerations of fairness and reciprocity (Graham et al. 2009).’

We performed GLMs to assess which questionnaire score significantly predicted our behavioural variables. Indeed, as the reviewer points out, we still had to perform 16 GLMs (8 variables OFF,PUN, OFFDIFF and PUNDIFF and associated RTs X 2 games UG and TP-DG), and to properly correct for multiple comparisons we should multiply our p-values by 16. We now state this in the results, keep the analyses only for the GLMs with the corrected p-values. We remove the correlation analyses that suffer even more from the multiple comparison problem (16X3 scales=48scales). We only keep post-hoc analyses that illustrate the findings of the GLMs (for figure 4).

Even though many of the mixed models results become close to significance/ not significant at all, we still report all the effects that were significant in the GLM, but now showing the corrected p-value and giving more cautious conclusions and interpretations. We believe that the questionnaire results, even though exploratory and not robust, are interesting and worse considering for future replications.

*In terms of the Rand re-analysis, what do the authors make of the fact that the difference did not appear between 1 and 2 defectors? More generally the logic of including the Rand re-analysis in this paper wasn’t as clear as it could be; more discussion of what it adds would be helpful.*

We hope to have clarified the logic of having this analysis. The idea is that the higher the number of participants violating norms by contributing with low amounts, the less this behaviour will be punished. So, being backed up by others in defection allows to protect from punishment. We added in the introduction:

‘In addition to these two adapted games, we re-analysed available data from a previous study ( Rand *et al.*, 2009) that involved a public goods game between four players with punishment to test whether the use of punishment changes with the number of people defecting. Similarly to playing in a group, we hypothesize that when several people violate norms, they may get away easier with it under the shield that ‘others did it too’. This allows to share responsibility for a punishable act, which thereby may become less prone to punishment.’

And in the results:

‘We tested our third prediction that inflicting punishment on unfair contributions will decrease with the number of people defecting. The reasoning here is that the more defectors on  a given round, i.e., the more people who violate the norm, the more their behaviour can be justified (they are not the only ones!) and therefore the more they can benefit from reduced punishment.’

We have also changed the way we choose the defectors in this re-analysis following the other reviewer’s comment, and the new results show a clearer linear decrease from 1 to 4 defectors (new figure 5). With our initial analyses, the results suggested that the decrease becomes significant only when there are enough defectors, i.e. 3, but not 2.

# Peer review: Reviewer response for version 1

**DOI:** 10.21956/wellcomeopenres.16925.r36622

**Published:** 2019-10-16

**Authors:** Justin W. Martin

**Affiliations:** 1 Department of Psychology, Boston College, Chestnut Hill, MA, USA

In this article, the authors investigate the influence of being in a group (vs. being an individual) on cooperation and punishment. In particular, they vary whether unfairness is a result of individual vs. group decision-making in the context of second-party punishment (indexed using the Ultimatum Game) and third-party punishment (indexed using the Third-Party Punishment variant of the Dictator Game). They find cooperation is lower when decisions are made by a group relative to when made by an individual. They find no overall punishment difference between group vs. individual decision-making, and find greater punishment as a second-party vs. a third-party. Finally, they find that reaction times are slower when deciding whether or not to punish a group (relative to punishing an individual), though they do not find that the decision to punish itself is slower when punishing a group.

 There are some things to like about this article. In particular, the topic is interesting and the authors have made their task and data publicly available.

 However, this article also has some major limitations.

 First, it is not clear that the authors’ main manipulation allows them to get at the principle mechanism they articulate. Specifically, the authors suggest that individuals may be less cooperative in the context of a group and punish groups more because group membership reduces responsibility (p. 2 first and third paragraphs). However, the authors do not assess perceptions of responsibility or include any additional manipulation that would directly target perceptions of responsibility. In the absence of any such mechanistic evidence, any effect of their manipulation could be due to other factors. In particular, one plausible alternative explanation focuses on practical considerations. That is, because the group’s offer was an average of the offers of the constituent members, any influence of being in a group (vs. being an individual) could simply be a function of the averaging that occurs to the offers. If I want to make a low offer as part of a group, I need to make my own offer especially low, so as to offset whatever offers the others may make. More generally, my point is that the authors have wished to interpret the lowered offers they find in the group condition as indicative of a willingness to violate norms in the context of group decision-making, but they do not provide any direct evidence in favor of that possibility.

 Second, the authors should do more to either qualify their interpretations or better justify them. For instance, they explain lower offers made as part of a group with the following (p. 7): “This suggests that participants were expecting less punishment when playing in a group as compared alone.” Do the authors have any data to suggest that participants’ expectations were the driving factor behind lower offers? In the absence of such evidence, these kinds of statements need to be qualified. A number of mechanisms could account for this difference (e.g. a different norm for offers in group vs. individual contexts; the strategic account I offered above; the responsibility focused account the authors proposed) and the authors need more evidence if they want to suggest that one is the primary factor.

 Third, the authors make predictions in the introduction about the relationship between reaction time and offers but do not explain or justify these predictions. More should be included here. In the absence of any explanation, these hypotheses feel post-hoc and unjustified. This is especially important given that these hypotheses were not pre-registered, there is no internal replication and many of these p-values hover around p = 0.05. Similarly, the authors should include more discussion of the scales they chose to include, as well as what their predictions for the relationship between these scales and their other data were. This is again especially important because these analyses were not pre-registered, were not replicated internally and sometimes hover around p = 0.05. And, the authors perform many analyses and comparisons using these scales, which would necessarily yield some significant results. In the absence of such justification, it would be better for the authors to describe these analyses as exploratory and caution their interpretation.

 Fourth, the authors should be more cautious in their discussion of their results. For instance, they write (p. 9): “there is a consistent slowing down to punish three individuals as compared to one individual”. This implies that punishment was slower in the group condition than the individual condition. However, this is not true: The authors found no relationship between the group manipulation and whether or not punishment was administered. They did find that responses (ignoring whether that response was to punish or not) were slower in the group condition. The way the authors describe this result, however, seems to indicate an effect that they did not find. Similarly, in the discussion they write (p. 11): “We importantly found that the punishment decisions were slower when punishers were faced with groups vs individuals, suggesting that it is also more time costly and less intuitive to choose whether to punish a group”. This follows the authors’ discussion of their overall finding of slower punishment vs. non-punishment. Again, readers will likely interpret this sentence as suggesting an effect that the authors do not find. When discussing these results, greater care needs to be taken in describing exactly which results were significant and which were not. Relatedly, the authors describe their reaction time results as suggesting that punishers (p. 11) “are more reluctant to punish a group vs. an individual”. Unless the authors have other evidence specifically implicit “reluctance”, they should be more cautious in interpreting their data. Slower reaction time could result from a number of factors (e.g. punishing a group may involve thinking about all 3 individuals impacted, slowing punishment relative to a condition in which only 1 person is impacted).

 Fifth, the authors note that they arrived at their sample size by selecting 40 participants per treatment and then multiplying by 4, yet they arrive at 150 participants (not 160). Was this perhaps a typo? If not, they should explain why they recruited 10 fewer participants than would make sense given their logic.

 I also have a number of more minor concerns.

 First, the authors interpret the slowed reaction time to punish vs. not punish as indicating that punishment is (p. 10) “more costly than not punishing”. Given the context of material costs paid and punishment itself (which is often discussed as being materially costly or non-costly), the authors should be clear earlier that they mean costly in terms of time. This is a somewhat non-standard meaning of costly and so clarifying it would be useful.

 Second, there are a decent number of typos/grammar issues (errors bolded; p. 3 : “To be protected against punishment, individuals delegate **decision** to others,...”; p. 3: “for an individual, being in a group could be a good way to reduce responsibility and thereby, the associated punishments for norm violations; p. 7: “To conclude on the proposer role, participants gave lower **offer** as a group vs alone“). I would encourage the authors to go over the paper carefully to correct these issues.

 Third, comparing directly to the data in Rand *et al*. (2009) ^1^ struck me as odd. There are likely many differences between that study and this one, and so it is not clear that the comparison is warranted. Furthermore, the analysis seems odd. When the authors say “We considered as defectors the players who gave less than the mean amount of contribution at each round”, what mean are they referring to? The mean for that round? This would then indicate that there were generally 2 defectors on every round (unless everyone contributed the same amount, e.g.). This seems like an arbitrary way of establishing what is considered defection. What about using half of the amount possible as a standard? And if the authors used a grand mean as the threshold, that seems problematic as well. For instance, if there was a group that was contributing below the grand mean, and yet all members were contributing the same, is it reasonable to call this defection? More generally, the relationship between responsibility and the number of defectors was not made totally clear.

 Fourth, in general I am not a fan of y-axis that do not include at least 1 anchor (ideally both should be included). In Figure 2A, the authors hone in on just the range from 3-4, only 10% of the entire scale. A more accurate scale would at least include the lowest value possible (0) but ideally would include the full range of the scale. Otherwise, the authors could be accused of restricting their y-axis to make their effect look larger.

 Fifth, in addition, in Figure 2A, the authors use two asterisks to mark the significance of the effect, which their caption describes as indicating p < 0.01. However, in-text they describe this difference as p = 0.025. This error should be corrected.

 Sixth, I would encourage the authors to pre-register their sample size, hypotheses and analysis plans in the future.

I confirm that I have read this submission and believe that I have an appropriate level of expertise to state that I do not consider it to be of an acceptable scientific standard, for reasons outlined above.

## References

[1] : Positive interactions promote public cooperation. *Science*.2009;325(5945) : 10.1126/science.1177418 1272-5 10.1126/science.1177418 19729661PMC2875121

# Response

**Published:** 2020-02-07

**Authors:** Marwa El Zein

**Affiliations:** University College London, UK

*In this article, the authors investigate the influence of being in a group (vs. being an individual) on cooperation and punishment. In particular, they vary whether unfairness is a result of individual vs. group decision-making in the context of second-party punishment (indexed using the Ultimatum Game) and third-party punishment (indexed using the Third-Party Punishment variant of the Dictator Game). They find cooperation is lower when decisions are made by a group relative to when made by an individual. They find no overall punishment difference between group vs. individual decision-making, and find greater punishment as a second-party vs. a third-party. Finally, they find that reaction times are slower when deciding whether or not to punish a group (relative to punishing an individual), though they do not find that the decision to punish itself is slower when punishing a group.*

*There are some things to like about this article. In particular, the topic is interesting and the authors have made their task and data publicly available.*

*However, this article also has some major limitations.*

We thank the reviewer for acknowledging the positive aspects of the paper, and for pointing out to major issues that we hope to have addressed.

*First, it is not clear that the authors’ main manipulation allows them to get at the principle mechanism they articulate. Specifically, the authors suggest that individuals may be less cooperative in the context of a group and punish groups more because group membership reduces responsibility (p. 2 first and third paragraphs). However, the authors do not assess perceptions of responsibility or include any additional manipulation that would directly target perceptions of responsibility. In the absence of any such mechanistic evidence, any effect of their manipulation could be due to other factors. In particular, one plausible alternative explanation focuses on practical considerations. That is, because the group’s offer was an average of the offers of the constituent members, any influence of being in a group (vs. being an individual) could simply be a function of the averaging that occurs to the offers. If I want to make a low offer as part of a group, I need to make my own offer especially low, so as to offset whatever offers the others may make. More generally, my point is that the authors have wished to interpret the lowered offers they find in the group condition as indicative of a willingness to violate norms in the context of group decision-making, but they do not provide any direct evidence in favor of that possibility.*

We agree with the reviewer that our experiment alone does not allow to exclusively explain our findings by the fact that being in a group reduces individual responsibility. Nevertheless, our design was driven by this hypothesis, and we now clarify this:

‘We note that while driven by our shared responsibility in groups hypothesis, our experimental design does not allow to characterize the exact mechanisms underlying differences in behaviour between norm violations and their punishment (and thereby be able to affirm that the effects are due to sharing responsibility solely relying on this experiment). However, to our knowledge, this is the first experimental design addressing whether these differences exist with such a controlled design, and directly comparing second and third-party punishment in repeated one-shot trials and a within-participant design.’

Moreover, we strongly believe that previous literature supports shared responsibility as a possible underlying mechanism for the behaviours we observe and now provide a more elaborate explanation why in the discussion section:

‘Our current finding that people are less generous in a group corroborates the idea that people in groups violate the norms more than alone given that (1) it replicates previous studies showing that individuals in groups display free-riding behaviours (Morgan & Tindale, 2002; Tindale & Kameda, 2017; Wildschut et al., 2003). These previous studies compared groups facing groups to individuals facing individuals and showed that groups are more competitive (Wildschut et al., 2003), defect more in a prisoner dilemma game ( Morgan & Tindale, 2002) and offer less in a joint decision in an ultimatum game (Bornstein & Yaniv, 1998). Our results complement these studies by showing that even when facing one individual, people are less generous if they are part of a group vs alone. (2) Interestingly, here, we show for the first time that this decreased generosity in group correlates with people’s overall generosity. Indeed, only those who gave low offers displayed a difference between playing in a group or alone. This shows that the group was compatible with the intention of those who were less sensitive to the norms and violated them more. (3) It has been suggested that increased defection in groups may relate to reduced ‘identifiability’ as a group, supporting the idea that people feel less ‘accountable’ when making selfish choices (Kugler et al. 2012). Backing this, the fact that groups of two members that could be easily identified playing a dictator game gave higher offers as a group (Cason and Mui, 1997) was linked to a reduced anonymity in such a context (Luhan et al. 2009). On the contrary, when anonymity is preserved in computer-based rather than face-to-face interactions, group members defect more than individuals. (4) Finally, groups that are procedurally interdependent (Wildschut et al. 2003) are less cooperative. This may be due to the fact that individual choices could not have been traced back, again allowing for anonymity and hiding behind the group (Kugler et al. 2012).’

Finally, we discuss other possibilities as the reviewer urged us to do, including the one given by the reviewer:

‘Despite substantial evidence that the effects observed in our study may be due to increased norm violation in groups because of shared responsibility, our experimental design does not allow us to disentangle the exact mechanism(s) underlying the observed reduction of offers in groups, and other explanations may as well be possible. For example, people may become more rational (Bornstein & Yaniv, 1998), and less biased as a group. They also may feel supported enough to act in a selfish, i.e. more greedy way (Schopler et al. 1995). In addition, one may argue that in our study, participants may have been basing their decision on the averaging procedure, by adjusting their offers to the expectations that others in the group will do. For example, a person who wants to give a low offer may lower their offer to offset the other group members offers. However, this last explanation is not consistent with the fact that only low offerors showed a difference between playing alone or in group as one would also expect those who want to give high offers to then adjust their behaviour by giving higher offers in groups (while they don’t). Finally, another possible explanation is that participants lowered their offers because of their expectation that other group members will also give low offers, given that predominantly unfair offers were presented in the context of the experiment (when participants were playing the receiver in the UG and the punisher in the TP-DG). However, the offer decrease for groups was already significant in the first round played in group as compared to the first round played individually, when they had less time to learn about the context they were playing in.’

*Second, the authors should do more to either qualify their interpretations or better justify them. For instance, they explain lower offers made as part of a group with the following (p. 7): “This suggests that participants were expecting less punishment when playing in a group as compared alone.” Do the authors have any data to suggest that participants’ expectations were the driving factor behind lower offers? In the absence of such evidence, these kinds of statements need to be qualified. A number of mechanisms could account for this difference (e.g. a different norm for offers in group vs. individual contexts; the strategic account I offered above; the responsibility focused account the authors proposed) and the authors need more evidence if they want to suggest that one is the primary factor.*

We agree with the reviewer and now suggest different possible interpretations of the results in the discussion. We added to the specific sentence mentioned here: ‘This *may* suggest that participants were expecting less punishment…’ as this sentence allowed us to do the transition to the punishment section, but do not elaborate further in the results section, in order to discuss different explanations in the discussion.

*Third, the authors make predictions in the introduction about the relationship between reaction time and offers but do not explain or justify these predictions. More should be included here. In the absence of any explanation, these hypotheses feel post-hoc and unjustified. This is especially important given that these hypotheses were not pre-registered, there is no internal replication and many of these p-values hover around p = 0.05. Similarly, the authors should include more discussion of the scales they chose to include, as well as what their predictions for the relationship between these scales and their other data were. This is again especially important because these analyses were not pre-registered, were not replicated internally and sometimes hover around p = 0.05. And, the authors perform many analyses and comparisons using these scales, which would necessarily yield some significant results. In the absence of such justification, it would be better for the authors to describe these analyses as exploratory and caution their interpretation.*

We apologize for not being clear about some of our predictions, we now better explain the motivations behind the hypotheses we have in the introduction:

‘Applying our key two hypotheses described above to the experimental paradigm, we predicted that 1) an individual in the group will offer less than the same individual alone, and make his/her decision faster because of less hesitation about violating the norms within a group, 2) the group will be punished less than the individual and punishment vs no punishment decisions facing a group will be slower reflecting a more hesitant choice, and 3) Inflicting punishment on unfair contributions will decrease with the number of people defecting. In an exploratory analysis, we collected self-reported scales to further investigate individual differences in norm violations and their punishment. Social value orientation was measured to link participants’ offering and punishment behaviours to a trait measurement of sharing with others ( Murphy et al., 2011). A psychopathy scale ( Paulhus & Williams, 2002) was collected to test the idea that higher psychopathy scores may be associated with lower influence of being in a group or punishing a group, as people with higher psychopathy scores may care less about being in a group. Finally, political identification was measured in order to test whether differences would appear in fairness attitudes in the context of playing alone or as a group, because liberals and conservatives have been associated with different considerations of fairness and reciprocity (Graham et al. 2009).’

We performed GLMs to assess which questionnaire score significantly predicted our behavioural variables. Indeed, as the reviewer points out, we still had to perform 16 GLMs (8 variables OFF,PUN, OFFDIFF and PUNDIFF and associated RTs X 2 games UG and TP-DG), and to properly correct for multiple comparisons we should multiply our p-values by 16. We now state this in the results, keep the analyses only for the GLMs with the corrected p-values. We remove the correlation analyses that suffer even more from the multiple comparison problem (16X3 scales=48scales). We only keep post-hoc analyses that illustrate the findings of the GLMs (for figure 4).

Even though many of the mixed models results become close to significance/ not significant at all, we still report all the effects that were significant in the GLM, but now showing the corrected p-value and giving more cautious conclusions and interpretations. We believe that the questionnaire results, even though exploratory and not robust, are interesting and worse considering for future replications.

  

*Fourth, the authors should be more cautious in their discussion of their results. For instance, they write (p.9): “there is a consistent slowing down to punish three individuals as compared to one individual”. This implies that punishment was slower in the group condition than the individual condition. However, this is not true: The authors found no relationship between the group manipulation and whether or not punishment was administered. They did find that responses (ignoring whether that response was to punish or not) were slower in the group condition. The way the authors describe this result, however, seems to indicate an effect that they did not find. Similarly, in the discussion they write (p. 11): “We importantly found that the punishment decisions were slower when punishers were faced with groups vs individuals, suggesting that it is also more time costly and less intuitive to choose whether to punish a group”. This follows the authors’ discussion of their overall finding of slower punishment vs.non-punishment. Again, readers will likely interpret this sentence as suggesting an effect that the authors do not find. When discussing these results, greater care needs to be taken in describing exactly which results were significant and which were not. Relatedly, the authors describe their reaction time results as suggesting that punishers (p. 11) “are more reluctant to punish a group vs. an individual”. Unless the authors have other evidence specifically implicit “reluctance”, they should be more cautious in interpreting their data. Slower reaction time could result from a number of factors (e.g. punishing a group may involve thinking about all 3 individuals impacted, slowing punishment relative to a condition in which only 1 person is impacted).*

As suggested here, we changed the vocabulary used to 1) clarify when the decision is the decision to punish or not to punish, and not only the ‘punishment’ decision as might have been implied. We changed the sentence to ‘…there is a consistent slowing down to make the decision of whether to punish or not three individuals as compared to one individual.’. We added in every place where it was needed: ‘punishment or no punishment decision’ and ‘punish or not punish’.

2) avoid incorrect interpretations of the slower RTs when facing groups by using ‘slowed down’ or ‘slower’ instead of ‘reluctance’.  

We agree that the slowing down when facing a group vs individuals may results from different factors. We had thought about the interpretation that the it might be due to thinking about who gave what and avoiding the punishment of fair participants trapped in group with unfair partners. We excluded that interpretation by showing that the slowing down was true even for very low offers, where the individual members offers were not ambiguous. We then suggest that given that the difference in reaction times is only present for low offers, participants seem to be slowed for groups vs individuals only when norms were violated (and only in the ultimatum game). We believe the explanation suggested by the reviewer that punishing a group may involve thinking about 3 individuals impacted (vs 1 in the individual condition) to be complementary to our conclusion: indeed, what the group offers is that the person punishing will take into account several people into account instead of one, and thereby be more hesitant to decide whether to punish or not the group. The fact that this only happens in second-party punishment and for low offers makes the effect specific to norm violations toward one self, rather than a general thinking about impacting 3 vs 1 person (or it should have been observed for all offers and in third-party punishment as well).

  

*Fifth, the authors note that they arrived at their sample size by selecting 40 participants per treatment and then multiplying by 4, yet they arrive at 150 participants (not 160). Was this perhaps a typo? If not, they should explain why they recruited 10 fewer participants than would make sense given their logic.*

We recruited 10 fewer participants (1 experimental session) because of practical issues related to timing and participants recruitment. As our sample size was based on a previous study using a different design, and our experiment design was novel, we do not believe having a few less participants than planned is highly problematic here. Indeed, most of our results were similar when the analyses were done on the first recruited group of 80 participants. We clarify in the methods:

‘Given our 4 conditions of interest (individual or group proposers in the proposer or the punisher role), we multiplied this number by 4 and tested 150 (instead of 160) participants because of practical issues related to timing and participants recruitment.’

*I also have a number of more minor concerns.*

**

*First, the authors interpret the slowed reaction time to punish vs. not punish as indicating that punishment is (p. 10) “more costly than not punishing”. Given the context of material costs paid and punishment itself (which is often discussed as being materially costly or non-costly), the authors should be clear earlier that they mean costly in terms of time. This is a somewhat non-standard meaning of costly and so clarifying it would be useful.*

**

We thank the reviewer for noting that using ‘costly’ for time in this context can be confusing. We decided not to use it and describe the slowing down effects as ‘less intuitive’ as more commonly described (Rand et al., 2012; Fehr & Fischbacher, 2004).

*Second, there are a decent number of typos/grammar issues (errors bolded; p. 3 : “To be protected against punishment, individuals delegate **decision **to others,...”; p. 3: “for an individual, being in a group could be a good way to reduce responsibility and thereby, the associated punishments for norm violations; p. 7: “To conclude on the proposer role, participants gave lower **offer **as a group vs alone“). I would encourage the authors to go over the paper carefully to correct these issues.*

We sincerely apologize for the errors and hope to have fixed them all.

*Third, comparing directly to the data in Rand et al. (2009) struck me as odd. There are likely many differences between that study and this one, and so it is not clear that the comparison is warranted. Furthermore, the analysis seems odd. When the authors say “We considered as defectors the players who gave less than the mean amount of contribution at each round”, what mean are they referring to? The mean for that round? This would then indicate that there were generally 2 defectors on every round (unless everyone contributed the same amount, e.g.). This seems like an arbitrary way of establishing what is considered defection. What about using half of the amount possible as a standard? And if the authors used a grand mean as the threshold, that seems problematic as well. For instance, if there was a group that was contributing below the grand mean, and yet all members were contributing the same, is it reasonable to call this defection? More generally, the relationship between responsibility and the number of defectors was not made totally clear.*

We are aware that there are differences between Rand et al. 2009 study and ours, but still believe that the re-analysis of the public good game with a punishment treatment is very relevant to our hypothesis that shared responsibility reduce punishment. We apologize for not making our reasoning clear enough and hope to have done so in our revised version of the manuscript. The idea is that the higher the number of participants violating norms by contributing with low amounts, the less this behaviour will be punished. So, being backed up by others in defection allows to protect from punishment. We added in the introduction:

‘In addition to these two adapted games, we re-analysed available data from a previous study ( Rand *et al.*, 2009) that involved a public goods game between four players with punishment to test whether the use of punishment changes with the number of people defecting. Similarly to playing in a group, we hypothesize that when several people violate norms, they may get away easier with it under the shield that ‘others did it too’. This allows to share responsibility for a punishable act, which thereby may become less prone to punishment.’

And in the results:

‘We tested our third prediction that inflicting punishment on unfair contributions will decrease with the number of people defecting. The reasoning here is that the more defectors on  a given round, i.e., the more people who violate the norm, the more their behaviour can be justified (they are not the only ones!) and therefore the more they can benefit from reduced punishment.’

On the how we established who defected:

We used the mean contribution per round to have a baseline for each group – because participants decide whether to punish after they see the contribution of each player. Our reasoning was that they may re-scale who they would want to punish based on what was the mean contribution at a given round. However, we understand that this may seem less intuitive (and possibly more problematic) than just sticking to the standard way, which is taking half the contribution as suggested by the reviewer.

We redid the analyses now considering the defectors as those who contributed less than 10, and the results similarly show the more defectors, the less use of punishment. With this way of calculating defectors, there are trials with 1 to 4 defectors (i.e. rounds where everyone is considered as defector).

*Fourth, in general I am not a fan of y-axis that do not include at least 1 anchor (ideally both should be included). In Figure 2A, the authors hone in on just the range from 3-4, only 10% of the entire scale. A more accurate scale would at least include the lowest value possible (0) but ideally would include the full range of the scale. Otherwise, the authors could be accused of restricting their y-axis to make their effect look larger.*

We have changed the figure as suggested including the full range of the scale.

*Fifth, in addition, in Figure 2A, the authors use two asterisks to mark the significance of the effect, which their caption describes as indicating p < 0.01. However, in-text they describe this difference as p = 0.025. This error should be corrected.*

We apologize for this error, it is now corrected.

*Sixth, I would encourage the authors to pre-register their sample size, hypotheses and analysis plans in the future.*

We agree with the reviewer that this should be done whenever possible and we will attempt to do that in the future.
